# Biogeographic Population Structure of Chimeric Blades of *Porphyra* in the Northeast Atlantic Reveals Southern Rich Gene Pools, Introgression and Cryptic Plasticity

**DOI:** 10.3389/fpls.2022.818368

**Published:** 2022-02-24

**Authors:** Elena Varela-Álvarez, Patrick G. Meirmans, Michael D. Guiry, Ester A. Serrão

**Affiliations:** ^1^CCMAR Centro de Ciências do Mar, CIMAR, Universidade do Algarve, Faro, Portugal; ^2^Institute for Biodiversity and Ecosystem Dynamics, University of Amsterdam, Amsterdam, Netherlands; ^3^AlgaeBase, Ryan Institute, National University of Ireland, Galway, Ireland

**Keywords:** red algae, plant chimera, polyploid, nori, biogeography

## Abstract

The genus *Porphyra sensu lato* (Bangiaceae, Rhodophyta), an important seaweed grown in aquaculture, is the most genetically diverse group of the Class Bangiophyceae, but has poorly understood genetic variability linked to complex evolutionary processes. Genetic studies in the last decades have largely focused on resolving gene phylogenies; however, there is little information on historical population biogeography, structure and gene flow in the Bangiaceae, probably due to their cryptic nature, chimerism and polyploidy, which render analyses challenging. This study aims to understand biogeographic population structure in the two abundant *Porphyra* species in the Northeast Atlantic: *Porphyra dioica* (a dioecious annual) and *Porphyra linearis* (protandrous hermaphroditic winter annual), occupying distinct niches (seasonality and position on the shore). Here, we present a large-scale biogeographic genetic analysis across their distribution in the Northeast Atlantic, using 10 microsatellites and cpDNA as genetic markers and integrating chimerism and polyploidy, including simulations considering alleles derived from different ploidy levels and/or from different genotypes within the chimeric blade. For *P. linearis*, both markers revealed strong genetic differentiation of north-central eastern Atlantic populations (from Iceland to the Basque region of Northeast Iberia) vs. southern populations (Galicia in Northwest Iberia, and Portugal), with higher genetic diversity in the south vs. a northern homogenous low diversity. For. *P. dioica*, microsatellite analyses also revealed two genetic regions, but with weaker differentiation, and cpDNA revealed little structure with all the haplotypes mixed across its distribution. The southern cluster in *P. linearis* also included introgressed individuals with cpDNA from *P. dioica* and a winter form of *P. dioica* occurred spatially intermixed with *P. linearis*. This third entity had a similar morphology and seasonality as *P. linearis* but genomes (either nuclear or chloroplast) from *P. dioica*. We hypothesize a northward colonization from southern Europe (where the ancestral populations reside and host most of the gene pool of these species). In *P. linearis* recently established populations colonized the north resulting in homogeneous low diversity, whereas for *P. dioica* the signature of this colonization is not as obvious due to hypothetical higher gene flow among populations, possibly linked to its reproductive biology and annual life history.

## Introduction

Ancient evolutionary lineages are more likely to retain traits and imprints of complex evolutionary histories that challenge the understanding of present population structures in extant species. An example is the case of the Bangiales, a distinctive order that represents an ancient lineage (e.g., Blouin et al., 2011) of morphologically simple red algae prone to complex genomic variability due to chimerism and mixoploidy (Varela-Álvarez et al., 2021). Fossil evidence supports this group as the oldest multicellular eukaryotes with sexual reproduction (Butterfield, 2000), and molecular dating indicates that they diversified approximately 250 million years ago from an origin along eastern Gondwanaland (current New Zealand and Australia), or from the Northwest Pacific (Yang et al., 2018), from where they spread worldwide (Xu et al., 2017). The species currently present in the North Atlantic have been hypothesized to originate from vicariant speciation driven by the Bering Strait (see Lindstrom and Cole, 1993; Bray et al., 2007; Kucera and Saunders, 2012; Mols-Mortensen et al., 2014). More specifically, *Porphyra umbilicalis* Kützing (Bangiaceae, Rhodophyta) from the Northwest Atlantic has been proposed as originating from Northeast Atlantic refugia by post-glacial colonization (Teasdale and Klein, 2010; Blouin and Brawley, 2012; Eriksen et al., 2016). Species of Bangiaceae (Bangiales, Rhodophyta) occur presently on intertidal and subtidal areas of coasts around the planet, visible as the gametophyte phase (n) of the life cycle, whereas their sporophytic phase (known as the “conchocelis phase,” 2n) consists of microscopic filaments that are extremely difficult to find in nature (Conway and Cole, 1977; Varela-Álvarez et al., 2018b). The gametophytes of some species form the most valuable seaweed aquaculture crop (mostly known as “nori” and with a retail value considered to be in excess of $1.3 billion per year, Blouin et al., 2011), which has been harvested and/or cultivated mostly in Japan, China and Korea for more than 1000 years.

Taxonomic complexity and cryptic diversity are widely reported in the family Bangiaceae. Recent taxonomic revisions (Sutherland et al., 2011; Sánchez et al., 2014) created multiple new genera and these and other molecular studies (e.g., Kucera and Saunders, 2012; Mols-Mortensen et al., 2012; Guillemin et al., 2016; Dumilag and Monotilla, 2018; Reddy et al., 2018) have revealed an unexpectedly high species diversity within the order Bangiales, currently numbering 186 species (Guiry and Guiry, 2021). Besides, in Sutherland et al. (2011) approximately half of 126 bladed samples included in the analysis were undescribed or uncertain. Beyond such studies at species level, their population structure and biogeography remain unclear below species level. Understanding population structure is an important tool to understand the conundrum (why so many species have evolved on so few morphological types) of cryptic diversity within the Bangiaceae, including inferring roles of hybridization, introgression, convergent evolution under similar selective pressures or historical events. Such processes can be inferred using a combination of molecular analyses across species geographical distributions.

Several challenges influence the population genetic structure in Bangiaceae, besides crypticity. Crypticity is a challenge for population studies because it is essential to distinguish each species to analyze population structure within species. In addition, some species display self-fertilization and asexual reproduction (Miura et al., 1979; Fujio et al., 1985, 1987; Gil-Kodaka et al., 1988). Another challenge is the presence of multiple alleles in supposedly haploid gametophytic blades, which can be influenced by divergent partial or complete chromosome copies (Fujio et al., 1988; Varela-Álvarez et al., 2018b,^2021^), in chimeric blades (Lindstrom, 1993; Varela-Álvarez et al., 2021) that retain multiple alleles from the conchospores that originated them.

The genus *Porphyra* (hereafter “*Porphyra sensu lato*” means all bladed taxa of the Bangiaceae”) is a chimeric system in multiple ways. When the sporophyte produces a conchospore, meiosis forms a tetrad consisting of the four meiotic products. These four cells develop together into a single leafy gametophytic thallus through successive mitotic divisions. Therefore, each gametophytic blade is composed of cells with different genetic compositions (Ohme and Miura, 1988).

Recently, using flow cytometry and microsatellite analyses, we found multiple genome sizes and multiple alleles at several loci in gametophytic blades of several *Porphyra* species from Portugal (Varela-Álvarez et al., 2018b). These results indicate the presence of several cytotype combinations, not only within populations, but also within individuals, where gametophytic blades can either have a single ploidy level or combined ploidies (mixoploids). Allopolyploids and diploidization have also been found in Asian species of *Porphyra* (e.g., Niwa and Sakamoto, 2010; Niwa et al., 2010, 2018; Zhong and Yan, 2020), and have been related to hybridization and introgression between closely related species (e.g., Niwa and Sakamoto, 2010; Niwa et al., 2010; Niwa et al., 2018). Hybridization and introgression have also been reported for species from the Northeast Atlantic (Mols-Mortensen et al., 2012). The detection of introgression, mixoploidy, chimerism and cryptic diversity was impeded by the previous lack of molecular markers allowing sufficient resolution at both the individual and population level for European species of *Porphyra*; these have only recently become available (Varela-Álvarez et al., 2017, 2018a), allowing high-resolution genotyping of large numbers of samples, for more progress in this direction.

The aim of the present study is to understand biogeographical patterns and infer hypothetical drivers of population genetic structure in two widely distributed *Porphyra* species from the Northeast Atlantic (from southern Portugal to Iceland) with contrasting mating systems and seasonality. *Porphyra dioica* J. Brodie and L. M. Irvine is dioecious, present all year on the coast and endemic to the Northeast Atlantic (type locality: Sidmouth, England; Brodie and Irvine, 2003; Brodie et al., 2008; Mols-Mortensen et al., 2012). *Porphyra linearis* Greville is a protandrous hermaphrodite species found only in winter, common on both sides of the North Atlantic (type locality is also in the Sidmouth area, England; Brodie and Irvine, 2003; Varela-Álvarez et al., 2004, 2005, 2007; Mols-Mortensen et al., 2012). We hypothesize that their population genetic structure may show evidence of hybridization and introgression as well as cryptic diversity, and that chimerism and polyploidy also shape the genetic composition of population of these species in the Northeast Atlantic.

## Materials and Methods

### Sampling and Species Identification

**FIGURE 1:**
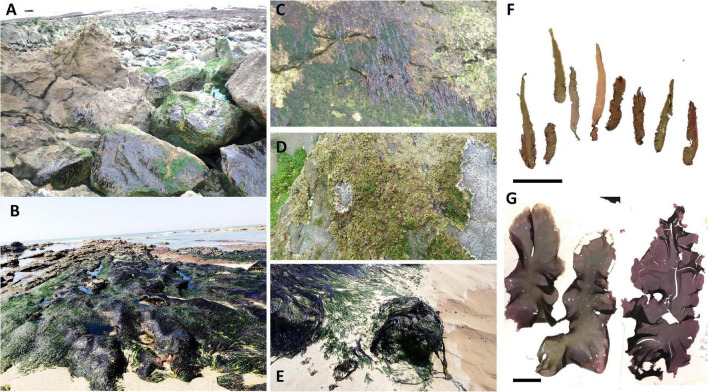
Photographs showing blades of *Porphyra* species used in this study in the field and in herbariums. **(A)** *P. lineari*s in Lisbon, Portugal, **(B)** *P. dioica* in Buarcos, **(C)** *P. linearis* in walls in Lisbon; **(D)** Winter *P. dioica* in Amado, **(E)** *P. dioica* in Galicia, **(F)** herbarium of the small winter form associated to *P. linearis* and/or the winter form of *P. dioica*, **(G)** herbarium samples of the annual lanceolate form associated with annual *P. dioica*.

Samples of *P. linearis* and *P. dioica* were obtained from 24 populations along the Northeast Atlantic coast including samples from populations from Portugal, Spain, France, Ireland and Norway (Table 1). Samples were collected fresh and brought to the laboratory to be examined for morphological and reproductive characters. Some samples were sent dried in silica gel by collaborators; such samples were re-hydrated to verify the morphology and reproduction. Species identification of the samples as *P. linearis* or *P. dioica* was done according to morphology (color, shape, holdfast, dimensions), reproduction, habitat, and seasonality (see Figure 1 and Table 2).

**TABLE 1:** Geographic origin and nuclear genetic diversity of populations of *P. linearis*, *P. dioica* and the winter form of *P. dioica* used in this study.

Species	Country	Population	Code	Lat Lon		N	EN As 2×	Hs As 2×	EN As 4×	Hs As 4×	EN As 8×	Hs As 8×
*Porphyra linearis*	Norway	Langesund	LAN	58.993	9.8295	24	1.66	0.29	1.61	0.29	1.61	0.29
	Ireland	Salthill	SAL	53.257535	–9.078936	9	2.08	0.37	1.98	0.39	1.99	0.38
	France	Roscoff	ROS	48.72692778	–3.98926667	18	2.11	0.33	1.81	0.32	1.81	0.32
	Spain	P Vasco	VAS	43.385825	–3.00245278	18	1.60	0.22	1.53	0.24	1.53	0.24
		Artabra	ART*	43.352906	–8.476037	18	2.38	0.37	2.29	0.35	2.28	0.35
		Baiona	BAI	42.124439	–8.847513	24	2.23	0.41	2.01	0.38	2.01	0.38
	Portugal	Moledo	MOL	41.83667222	–8.87438333	22	2.24	0.34	2.18	0.43	2.19	0.43
		Bartolomeu	BAR	41.57434167	–8.80002778	22	2.84	0.48	2.76	0.45	2.83	0.45
		Peniche	PEN	39.36131111	–9.34876944	24	2.03	0.34	1.89	0.31	1.89	0.31
		Raso	RAS*	38.710123	–9.486064	16	2.22	0.44	2.08	0.42	2.08	0.42
		Belém	BEL**	38.69138889	–9.21597778	29	2.12	0.41	1.88	0.35	1.87	0.35
*Porphyra dioica*	Ireland	Spiddal	SPI	53.24408	–9.298322	18	1.76	0.32	1.73	0.34	1.73	0.34
		Salthill P2	SAL2	53.257599	–9.078944	9	1.57	0.23	1.58	0.28	1.58	0.28
	France	Malo	MAL	48.65125	–2.026861	4	1.43	0.06	1.38	0.19	1.38	0.19
	Spain	Herminia	HER	43.3874	–8.39285	11	2.24	0.48	2.11	0.45	2.09	0.45
		Artabra P2	ART2	43.352779	–8.478103	15	1.99	0.36	1.87	0.33	1.87	0.33
		Esteiro	EST	42.789556	–8.970887	13	2.23	0.48	2.05	0.44	2.05	0.44
		Sansenxo	SAN	42.397501	–8.792579	12	1.90	0.36	1.83	0.36	1.86	0.37
		Sansenxo P2	SAN2	42.394723	–8.769136	17	2.29	0.46	2.08	0.41	2.06	0.41
	Portugal	Gelfa	GEL	41.797328	–8.873442	19	2.16	0.37	2.02	0.36	2.00	0.36
		Buarcos	BUA**	40.17196	–8.893759	38	2.20	0.43	2.08	0.40	2.08	0.40
		Oeiras	OEI	38.68555	–9.309167	26	1.76	0.33	1.72	0.31	1.72	0.31
Winter *P. dioica*	Spain	Patos	PAT	42.154873	–8.825847	10	2.28	0.32	1.96	0.35	1.96	0.35
	Portugal	Amado	AMA	37.16191389	–8.90590556	22	2.03	0.40	1.97	0.40	1.97	0.40

Lat/Lon in decimal degrees, N: Number of individuals genotyped. EN: Effective number of alleles. Hs: Heterozygosity within populations, all calculated considering the data as diploid 2×, tetraploid 4× or octoploid 8×. When considering the data as 4× or 8×, the maximum likelihood-correction for polyploidy dosage is applied. *Calculations after excluding individuals from winter P. dioica mixed in the population (2 individuals in Cabo Raso and 1 individual in Artabra). **Data from 2 populations from Varela-Álvarez et al. (2018b).

**TABLE 2:** Morphological, ecological, reproductive and genetic characters of the species used in this study.

			*Porphyra linearis*			*Porphyra dioica*	
			
		First description *Greville, 1830*	*Varela Álvarez, 2002*	This study	First description *Brodie and Irvine, 1997*	This study	*Winter form* This study
FORM	Dimensions	3–5 inch (7–12 cm) length ½ inch (1.5 cm) width	2–15 cm length 0.5–3 cm width	2–15 cm length 0.5–3 cm width	Up to 70 cm length Up to 29 cm width	10–50 cm length 5–15 cm width	2–20 cm length 0.5–3 cm width
	Color	Reddish – purple	Dark red to reddish Light brown	Dark red to reddish Light brown	Dark Purple to dark green	Dark Purple to dark green	Dark red to reddish – brown
	Shape	Linear or linear – lanceolate. Margin slightly Waved	Linear to pear shaped on the base, slender plants	Linear to pear shaped on the base, slender plants	Lanceolate	Lanceolate	Linear to pear-shaped on the base
	Holdfast	Very minute disk	Holdfast clearly separated from the thallus at the base	Holdfast clearly separated from the thallus at the base	Holdfast clearly separated from the thallus at the base	Holdfast clearly separated from the thallus at the base	Holdfast clearly separated from the thallus
NICHE	Habitat	On rocks and stones, high water mark	On rocks, high littoral, very wave exposed area	On rocks, high littoral, very wave exposed area, in patches	Lower shore, usually in boulders in sand	Lower shore, usually in boulders in sand	On rocks, high littoral, very wave exposed area, in patches alone, or in patches mixed with *P. linearis*
	Seasonality	Annual. April and May	October to May	October to May	All year	All year	December to March
REPRODUCTION	Reproduction	Oval granules, not arranged in a quaternate manner	Mainly monoecious, sometimes dioecious, probably sequentially protandrous	Mainly monoecious, but sometimes dioecious, probably sequential protandrous	Dioecious, reproductive sori marginal, occasionally monoecious	Dioecious, reproductive sori marginal, occasionally monoecious	−
	Reproduction bodies	Partly scattered, partly in lines	Marginal. Male sorus pale yellow edge. Female sorus: red edge on the base	Marginal. Male sorus pale yellow edge. Female sorus: red edge on the base	Marginal yellow and reddish female sori	Marginal. Strong yellow and reddish female sori	−
DNA	*Rbc*L Haplotypes	−	−	A, B, C, E Introgressed D, F	−	F, G, H, I, J, K	F
	Genotypes	−	−	*P. linearis*, hybrids × *P. dioica*	−	*P. dioica*	*P. dioica*

*Data from different sources and in reference to the original descriptions.*

### DNA Extraction, Polymerase Chain Reactions Amplification and Genotyping

Genomic DNA was isolated from 238 *P. linearis* individuals and 152 *P. dioica* individuals using the LiCl extraction protocol described by Hong et al. (1992) as modified by van Oppen et al. (1995). Polymerase chain reaction (PCR) for 10 selected microsatellite markers (Varela-Álvarez et al., 2017, 2018a; Supplementary Table 1) were performed separately for each locus in a 20 μl reaction volume containing 5–50 ng genomic DNA. Amplifications were conducted following the PCR programs and conditions as described in Varela-Álvarez et al. (2017, 2018a) using a GeneAmp 2720 thermal cycler (Applied Biosystems). Amplified fragments were separated electrophoretically using an ABI PRISM 3130xl (Applied Biosystems) automated capillary sequencer at CCMAR, Portugal, and sized with GeneScan-350ROX size standard (Applied Biosystems). In addition, genotype data from samples from two locations previously used in Varela-Álvarez et al. (2018b) was added to the data set: 22 genotypes from *P. linearis* from Belém and 30 genotypes from *P. dioica* from Buarcos, both locations in Portugal. Alleles were scored in GENEMAPPER v.4.1 (Applied Biosystems). Binning and allele rounding were checked with GENEMAPPER v.4.1 and TANDEM (Matschiner and Salzburger, 2009) for the full data set containing 442 multilocus genotypes (Supplementary Appendix 1).

### Data Sub-Sample to Address Chimerism and Polyploidy

**FIGURE 2:**
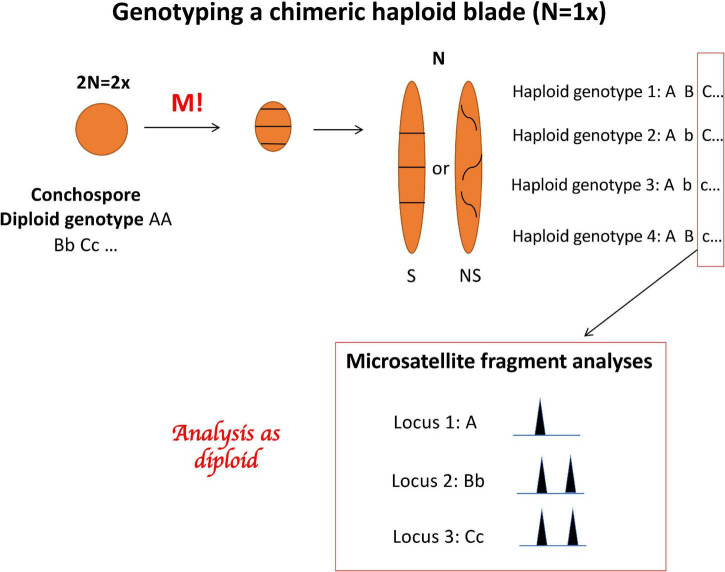
*Porphyra* life cycle showing the development of the conchospore into a chimeric haploid blade with 4 genotypes spreading along the blade, and the genotyping with three microsatellite loci displaying a multiple allele genotypes in the haploid blade.

In *Porphyra*, the gametophytic thallus is a chimera that develops directly from the four meiotic products and therefore contains different genotypes that are arranged either in discrete sections or distributed haphazardly across the blade (Figure 2). Moreover, *Porphyra* blades can also contain several rounds of genome duplications within the same thallus representing either different ploidy levels in the same individual (mixoploids, Varela-Álvarez et al., 2018b,^2021^) or different cytotypes within the same population (e.g., diploid (2×), tetraploid (4×) individuals, etc., see Niwa and Sakamoto, 2010; Niwa et al., 2010, 2018; Varela-Álvarez et al., 2018b). Therefore, finding multiple alleles in a thallus genotype could be either the result of chimerism (different genotypes derived from the tetrad) and/or different ploidy levels. Therefore, neither the true dosage of the alleles nor the true ploidy level in the sampled tissue can be known, which complicates and possibly biases the analysis of the genetic data (Meirmans et al., 2018). To address these issues and to explore the extent of a possible bias, we performed the analyses with multiple assumed ploidy levels for the whole dataset. We created three different versions of the genetic datafile, with the data coded as 2×, 4×, and 8×, subsampling alleles within individuals when necessary (up to 6 alleles per locus were observed; see Supplementary Appendix 1). We started the analyses assuming a ploidy level of 2×, because it is the most basic scenario; when genotyping a chimeric haploid thallus (a blade where all the cells have one set of chromosomes), up to 2 alleles per locus can be found because the sporophyte producing the tetrad would then be diploid (Figure 2). Correspondingly, for the 4× coding –representing the case of a chimeric diploid thallus– up to 4 alleles can be found per locus. Finally, the 8× coding represents a chimeric tetraploid thallus.

### Nuclear Genetic Diversity and Genetic Differentiation

Two summary statistics of nuclear genetic diversity were calculated for every population in each species using GENODIVE 3.04 (Meirmans, 2020): *EN*, the effective number of alleles (the number of equally frequent alleles it would take to achieve a given level of gene diversity) and *H*_S_, the expected heterozygosity using the Hardy-Weinberg equilibrium (also known as Gene Diversity) (Table 1). Each index was calculated considering the data as diploid (2×), tetraploid (4×), and octoploid (8×), while applying the maximum likelihood-correction for unknown allele dosage (De Silva et al., 2005) implemented in GENODIVE, for the scenarios where data were considered as polyploid (4× or 8×). When individuals had more unique alleles than possible under these ploidy-scenarios, a random subsample of the alleles was drawn. Since we did not know the exact ploidy of the samples and we did not have any information of the dosage of the alleles, we were not able to calculate the inbreeding coefficient *F*_IS_.

Genetic population structuring and admixture was evaluated with the software STRUCTURE v. 2.3.2.1 (Pritchard et al., 2000). STRUCTURE was run with the admixture model with the assumed number of clusters (*K*) ranging from 1 to 25. For each value of *K*, ten replicate runs were carried out consisting of 100,000 Markov Chain Monte Carlo (MCMC) iterations after a burn-in period of 10,000, without any prior information on the species or population of the sampled individuals. The optimal number of clusters was determined using the **Δ***K* statistic (Evanno et al., 2005). For this optimal value of *K*, we chose the replicate with the highest overall likelihood for graphical presentation; the bar chart was created in Microsoft Excel. Extraction of the membership coefficients and calculation of the **Δ***K* statistics were done using a custom script designed to run STRUCTURE using R (Supplementary Appendix 2). Separate analyses were done considering the data as diploid 2×, tetraploid 4×, and octoploid 8×. Pairwise differentiation among all pairs of populations was calculated by Nei’s Gst and JostD coefficients.

In addition to the STRUCTURE analysis, we also studied the main patterns in the genotypic differentiation by a Principal Components Analysis (PCA) based on the allelic variation across all genotyped individuals using GENODIVE 3.04 (Meirmans, 2020). The PCA was performed three times, considering the data as diploid 2×, tetraploid 4×, and octoploid 8×, as described above.

The strength of the genetic differentiation within and among the clusters recognized by STRUCTURE was estimated using a series of Analyses of Molecular Variance (AMOVA, Excoffier et al., 1992). The first AMOVA quantified the strength of the differentiation among the two species, *P. dioica* and *P. linearis*; where we used the STRUCTURE assignment to the two species (*K* = 2) rather than the morphological species identification, with populations grouped within species. We then proceeded to quantify the differentiation within each species separately, based on the main within-species clusters. For these latter analyses, we removed three individuals that were identified genetically as *P. dioica* in populations where all other individuals were identified genetically as *P. linearis*. No significance test was performed for these AMOVAs as that would lead to circularity in reasoning (Meirmans, 2015). Despite the lack of testing, the AMOVAs are very informative as STRUCTURE does inform about the presence of population structure, but not about the strength of the clustering. All AMOVAs were performed three times: for the assumed ploidy levels of 2×, 4×, and 8×.

### Sequencing and Phylogenetic Analyses

A selection of 2 to 6 individuals was made from each sampling location for amplification and sequencing of the cpDNA rbcL-region, following the methods by Broom et al. (2010) with primers KitoF1 (5′-ATG TCTCAATCCGTAGAATCA-3′), and JrSR (5′-AAGCCCCTTGTGTTAGTCTCAC-3′). The following amplification profile was used for the PCR-amplification of the region: 1 cycle of 94°C for 5 min; 35 cycles of 94°C for 30 s, 50°C for 30 s min, 72°C for 1.30 min; 1 cycle of 72°C for 10 min. Sequences were also retrieved from GenBank from additional locations from Britain and Iceland for *P. dioica* and from Britain, Iceland, and the United States for *P. linearis*. As outgroup, sequences from *Pyropia yezoensis* were downloaded from GenBank (accession numbers: MG604384 and AB455543). The resulting sequences were edited to eliminate ambiguities and aligned with GENEIOUS 6.1.6 (Biomatters Ltd., Auckland, New Zealand) and MEGA X (Kumar et al., 2018; Stecher et al., 2020). The final data set contained 73 sequences, with a region of 1141 bp shared among all sequences including the outgroups. The frequency of the different rbcL haplotypes for each location were plotted to visualize their geographical pattern. The phylogenetic relationships among the haplotypes were examined using a haplotype network and phylograms. The haplotype networks were drawn in POPART^1^ using the TCS (Clement et al., 2002) network approach (95% parsimony connection limit). The phylograms were constructed using Maximum likelihood (ML) methods with MEGA X software. Robustness of the trees was tested using 10,000 bootstrap replicates. In addition, the number and delimitation of rbcL phylogroups was visually compared for congruence with the multilocus genotypic clusters found in the STRUCTURE analysis and also in the Principal Components Analysis.

## Results

### Species Distinction, Cryptic Morphotype and Admixture

**FIGURE 3:**
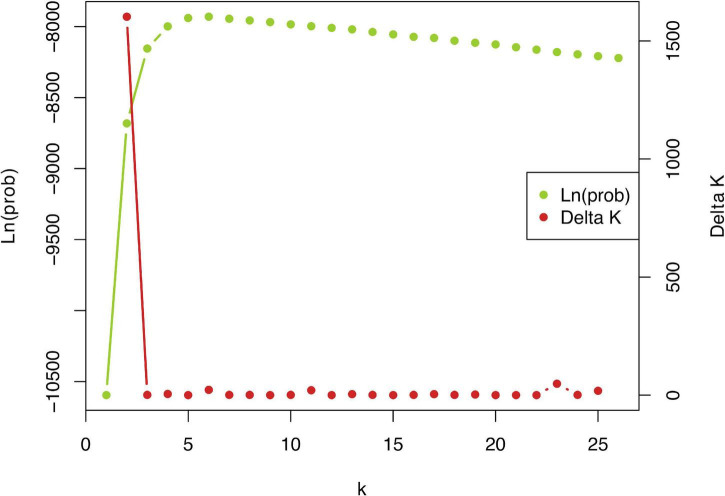
Plot of ΔK statistic of Evanno et al. (2005) detecting the number of K groups that best fit the data (best *K* = 2).

**FIGURE 4:**
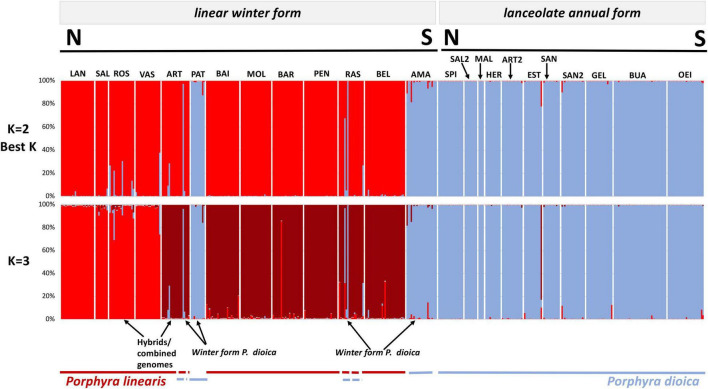
Genome constitution of the 442 genotypes inferred by STRUCTURE to 2 and 3 inferred clusters based in 10 microsatellite loci. A column represents each individual; different colors within columns indicate the maximum likelihood probability of belonging to different clusters. Blue colors are associated to *P. dioica* (either winter or annual) nuclear genomes and red colors are associated to *P. linearis* nuclear genomes. Population codes in Table 1.

**FIGURE 5:**
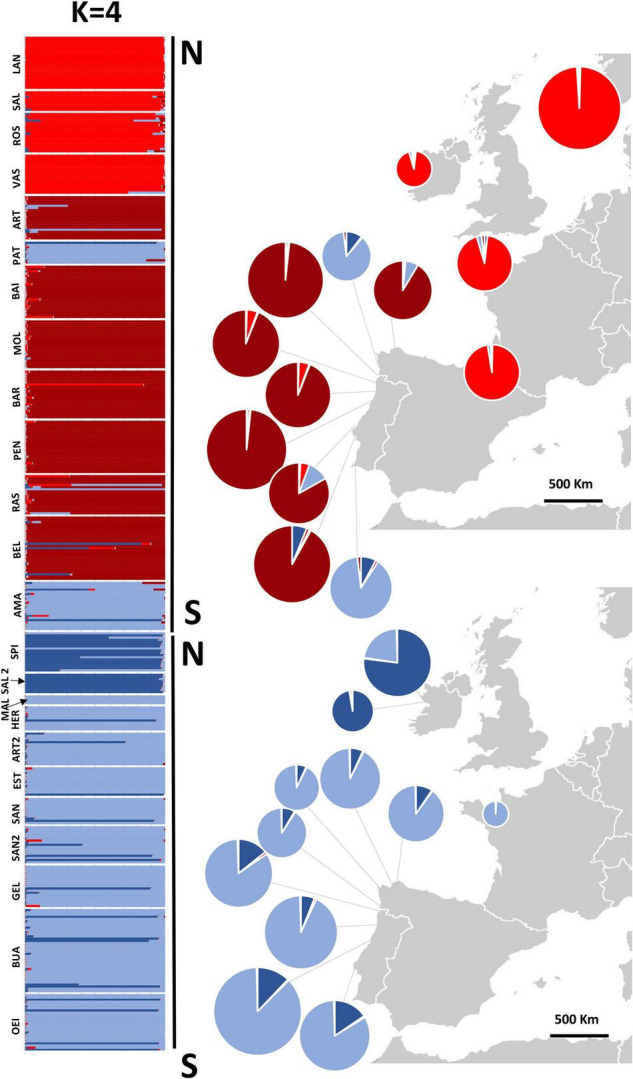
Structure assignment of individuals to 4 inferred clusters and pie charts of combined genetic ancestries of all individuals sampled in each population. Pie charts on the right give the mean ancestry estimates for each population across 10 replicates. Population codes in Table 1.

**FIGURE 6:**
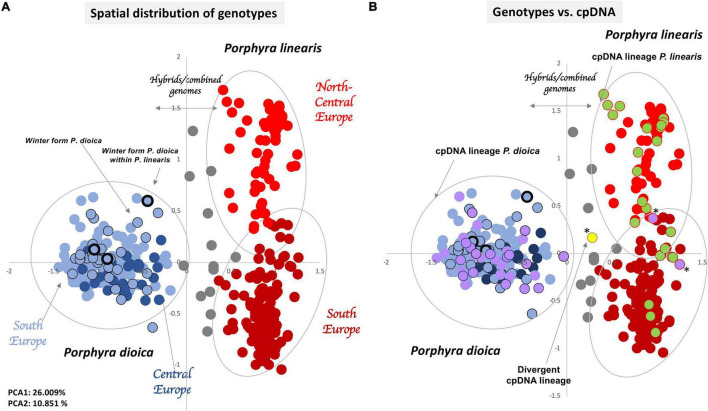
Spatial representation of genetic differentiation of the 442 genotypes. Principal component analyses (PCA) based on allelic variation at 10 loci. **(A)** PCA with *Porphyra linearis* genotypes in red (north Europe light red, south Europe dark red), *Porphyra dioica* genotypes in blue (central Europe dark blue, south Europe light blue), Winter form of *P. dioica* in blue surrounded by a black circle and hybrids or genotypes with combined genomes in gray. **(B)** PCA comparing genotypes vs. cpDNA rbcL sequences for each genotype found, with genotypes having rbcL sequences within the cpDNA lineage of *P. linearis* in green, genotypes having rbcL sequences with the cpDNA lineage of *P. dioica* in purple, and one genotype with a rbcL sequence within the cpDNA lineage of *Porphyra* spp. (*) Introgressed individuals.

STRUCTURE analysis of all 442 genotypes showed an optimum at *K* = 2 according to the **Δ***K* criterion (Figure 3), at which the cluster assignments generally aligned with the *a priori* species delimitations into *P. linearis* and *P. dioica*. Surprisingly, however, the STRUCTURE results also indicated the cryptic presence of *P. dioica* genotypes within samples originally attributed to *P. linearis*. These individuals had the morphology (shape, color, size) and winter seasonality of *P. linearis* but *P. dioica* genotypes (both the nuclear genomes or chloroplasts genomes, see below). This new cryptic morphotype was found either as a few individuals intermixed within *P. linearis* populations (Artabra and Cabo Raso) or as full populations (Patos and Amado) (Tables 1, 3 and Figures 4–6) but these appear to be restricted to southern Europe. The PCA (Figure 6), showed similar results as STRUCTURE, with *P. linearis* genotypes grouped together and clearly distinguished from the *P. dioica* genotypes, and showed also the same cryptic samples clustering to *P. dioica*. We refer to this new cryptic form the “winter form” of *P. dioica* from here on. For the further analyses reported below, we either included them as a separate third taxon or included them with the southern populations of *P. dioica* (indicated in the Tables).

**TABLE 3:** Summary of genetic diversity of populations of *P. linearis*, *P. dioica* and the winter form of *P. dioica* by geographical areas.

	*N*	*EN*	*H*_S_	Detected nuclear hybrid	Detected rbcL Haplotype	Detected rbcL introgression
PL North	69	1.77 (0.30)	0.30 (0.09)	Yes	A	No
PL South	156	2.14 (0.39)	0.40 (0.09)	Yes	A,B,C,D E,F	*P. dioica*
PD North	31	1.52 (0.19)	0.21 (0.09)	No	F,G,H	No
PD South	151	1.96 (0.27)	0.41 (0.07)	No	F,G,I,J,K	No
PD South + WPD	186	1.79 (0,20)	0.35 (0.08)	No	F,G,I,J,K	No
Winter PD	35	1.6 (0.18)	0.16 (0.13)	Yes	F	No
PL	225	1.88 (0.32)	0.36 (0.07)	Yes	A,B,C,D E,F	*P. dioica*
PD	182	1.79 (0.22)	0.36 (0.07)	No	F,G,H,I,J,K	No
PD + Winter PD	217	1.72 (0.19)	0.32 (0.08)	Yes	F,G,H, I, J, K	No
All	442	1.78 (0.21)	0.36 (0.07)	Yes	A,B,C,D E,F,G,H,I, J, K	Yes

*N: Number of individuals genotyped. EN: Effective number of alleles (the number of equally frequent alleles it would take to achieve a given level of gene diversity); H_S_: Heterozygosity within populations or Gene diversity (the expected frequency of heterozygotes within subpopulations, assuming Hardy-Weinberg equilibrium). In brackets the standard deviation is shown. All calculated considering the data as diploid (2×). In addition, in the rightmost columns, the rbcL haplotypes detected in each area and species, and the inferred haplotype introgression are given.*

In addition to the cryptic winter form, both STRUCTURE and the PCA showed the presence of several admixed genotypes that are presumably hybrids between the two species. All above results were the same when considering species as diploid (Figures 3–6), tetraploid, or octoploid (Supplementary Figures 1–4).

### Population Structure Between North and South of Europe From Nuclear Markers

Inferring sub-group genetic structure using STRUCTURE, higher levels of *K* showed a division into within-species groups. For *K* = 3, *P. linearis* was split into two clusters (Figure 4 top; two shades of red), corresponding to North Central (Norway, Europe, Ireland, Britain, France and North Central Spain) vs. South Europe (including Northwest Spain and Portugal). *K* = 4 leads to an additional splitting of *P. dioica* genotypes, where the two populations from Ireland were separated from the southern populations (Figure 5; two shades of blue). However, for *P. dioica* there was admixture between the two clusters across most populations. The individuals with the cryptic winter form of *P. dioica* were assigned to the South Europe cluster, also with some admixture. Similar divisions into clusters and similar patterns of admixture were obtained when STRUCTURE was run for each species separately (results not shown).

For both species, populations from the South Europe STRUCTURE cluster (Spain and Portugal) showed a higher number of alleles, and higher gene diversity (Tables 1, 3) and were more differentiated in comparison with populations from the northern cluster (Table 3 and Supplementary Table 2). For *P. linearis*, intrapopulation genetic diversity (either measured as expected heterozygosity or as effective number of alleles) was highest in the North of Portugal. For *P. dioica* there was less spread in the genetic diversity and the most diverse population differed, depending on the summary statistic and the assumed ploidy level.

Despite the presence of some admixed individuals, there was a strong genetic differentiation between the two species, as shown by the AMOVAs. The *F*_CT_ statistic that quantifies the differentiation among the two species had values of 0.26, 0.29, and 0.31, for the assumed ploidy levels of 2×, 4×, and 8×, respectively (see Table 4). The relatively small difference between these values indicates that the assumed ploidy level has limited impact on the estimation of the genetic divergence.

**TABLE 4:** Strength of the genetic population structure as estimated using an AMOVA.

			As 2×		As 4×		As 8×	
	Source of Variation	*F*-stat.	*F*-value	%var	*F*-value	%var	*F*-value	%var
Among Species	Within Individuals	*F*_IT_	0.38 (0.16)	61.7	0.58 (0.11)	41.7	0.68 (0.1)	32.2
	Among Individuals within Pops.	*F*_IS_	0.01 (0.12)	0.5	0.29 (0.09)	17.0	0.41 (0.09)	22.3
	Among Pops. within Species	*F*_SC_	0.16 (0.02)	11.8	0.18 (0.03)	12.7	0.21 (0.03)	14.5
	Among Species	*F*_CT_	0.26 (0.11)	26.0	0.29 (0.12)	28.6	0.31 (0.13)	31.0
*P. linearis* North vs. South	Within Individuals	*F*_IT_	0.32 (0.12)	67.5	0.55 (0.08)	45.2	0.65 (0.07)	35.4
	Among Individuals within Pops.	*F*_IS_	0.01 (0.12)	0.8	0.31 (0.08)	20.4	0.43 (0.08)	27.1
	Among Pops. within Clusters	*F*_SC_	0.1 (0.01)	8.0	0.12 (0.01)	8.6	0.13 (0.01)	9.4
	Among Clusters	*F*_CT_	0.24 (0.06)	23.6	0.26 (0.06)	25.8	0.28 (0.07)	28.1
*P. dioica* North vs. South	Within Individuals	*F*_IT_	0.2 (0.21)	80.5	0.43 (0.16)	57.5	0.55 (0.15)	45.4
	Among Individuals within Pops.	*F*_IS_	0.01 (0.16)	0.5	0.27 (0.13)	21.0	0.38 (0.13)	28.0
	Among Pops. within Clusters	*F*_SC_	0.05 (0.02)	4.5	0.07 (0.03)	5.7	0.09 (0.04)	7.6
	Among Clusters	*F*_CT_	0.15 (0.12)	14.6	0.16 (0.13)	15.8	0.19 (0.14)	19.0
*P. dioica* Morphotypes	Within Individuals	*F*_IT_	0.09 (0.17)	91.1	0.35 (0.13)	65.4	0.47 (0.13)	52.7
	Among Individuals within Pops.	*F*_IS_	0.01 (0.16)	0.6	0.27 (0.13)	23.9	0.38 (0.13)	32.6
	Among Pops. within Morphs	*F*_SC_	0.09 (0.03)	9.3	0.11 (0.04)	11.0	0.14 (0.04)	14.0
	Among Morphotypes	*F*_CT_	−0.01 (0.01)	−1.0	0.00 (0.02)	−0.3	0.01 (0.03)	0.6

*Values of the F-statistics corresponding to the different hierarchical levels of the population structure, and the associated percentages of the total genetic variance (%var). The population structure was based on the results of the STRUCTURE analyses (Figures 4, 5), both for delimiting the two species, and for assigning populations to the North and South clusters within species. To avoid circularity, no P-values were calculated (Meirmans, 2015). Following the Structure results, the cryptic Winter form of P. dioica was included in the southern cluster of that species, except for the final AMOVA. There, only P. dioica was included where one cluster was composed of the Winter form and a second cluster was composed of all other populations, in order to test for possible differentiation between the two morphotypes.*

In both species, STRUCTURE revealed a division of the populations into northern and southern clusters, but the degree of divergence (*F*_CT_) between the clusters was stronger for *P. linearis* than for *P. dioica*, irrespective of the assumed ploidy level (see Table 4). Interestingly, the differentiation among population within clusters (*F*_SC_) was also stronger in *P. linearis*. When *P. dioica* was split into two clusters representing the two morphotypes (the Winter form and the annual form), no differentiation was found according to *F*_CT_ with values of −0.01, 0.00, and 0.01 for the assumed ploidy levels of 2×, 4×, and 8×.

### cpDNA Phylogeographic Analyses

**FIGURE 7:**
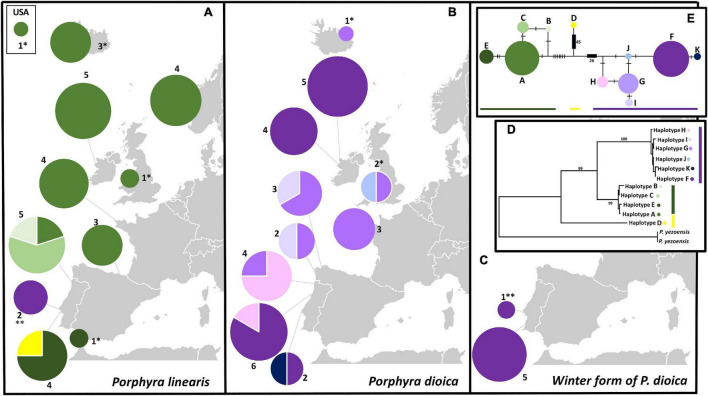
Geographical distribution and phylogenetic relationships of rbcL haplotype sequences used in this study; **(A)** Geographical distribution of pie charts for *P. linearis* along the biogeographical range on the Northeast Atlantic (including a site from United States); **(B)** Geographical distribution of pie charts for *P. dioica* along its full biogeographical range, **(C)** Geographical distribution of pie chart for the winter form of *P. dioica* found in this study; (For all, the color key for the haplotypes found is on the right part of the figure); **(D)** Phylogenetic phylogram by ML based on the 11 haplotypes (plus 2 outgroups) obtained in 71 sequences of 1141 nucleotides; numbers represent bootstrap values for the consensus tree for each analyses. The evolutionary history was inferred by using the Maximum Likelihood method (ML), based on the Tamura-Nei model Tamura and Nei (1993), **(E)** TCS network of the 71 rbcL sequences, 9 extracted from Genbank, and 62 produced in this study. Size in each pie chart is proportional to the number of sequences that belong to one haplotype. Mutations represented by hatch marks or numbers in brackets.

CpDNA analyses based on 71 sequences (62 produced in this study, Table 5), with 1141 aligned positions and 11 distinct haplotypes, also supported the separation of the samples into two distinct clades coincident with *P. linearis* and *P. dioica*. Within each species, the haplotypes were generally closely related, with only a few mutations steps between haplotypes, with the exception of one divergent haplotype (Haplotype D) found within the *P. linearis* samples. This could be the case of a recombinant haplotype, or a lineage from an unknown cryptic species, at a introgressed or heteroplasmic individual but further research is needed to clarity this aspect. Haplotype F was widespread in *P. dioica* but was also found in two *P. linearis* individuals from one site (Cabo Raso) in the South Europe cluster, where the microsatellites had shown admixture for those two exact individuals. The winter form of *P. dioica* only displayed this haplotype F, agreeing with the results from the microsatellites (Figures 6, 7).

**TABLE 5:** Sample localities for rbcL haplotyping.

	Country	Locality	N	Haplotype	Accession Number	Source (Population code)
*P. linearis*	United States	Maine	1	Hpl A	JN028945	Kucera and Saunders, 2012
	Iceland	Hofnin, Gardi	3	Hpl A	JN787103; JN787104; JN787105	Mols-Mortensen et al., 2012
	Norway	Langesund	4	Hpl A	OL477717, OL477718, OL477719, OL477720	This study (LAN)
	Britain	Aberystwyth	1	Hpl A	HQ687547	Sutherland et al., 2011
	Ireland	Salthill	5	Hpl A	OL477712, OL477713, OL477714, OL477715, OL477716	This study (SAL)
	France	Roscoff	4	Hpl A	OL477708, OL477709, OL477710, OL477711	This study (ROS)
	Spain	Sopelana, Basque	3	Hpl A	OL477705, OL477706, OL477707	This study (VAS)
	Portugal	Bartolomeu	5	Hpl A; B; C	OL477700, OL477701, OL477702, OL477703, OL477704	This study (BAR)
		Raso^ + +^	2	Hpl F	OL477693, OL477694	This study (RAS)
		Belém	4	Hpl D, E	OL477696, OL4776967, OL477698, OL477699	This study (BEL)
	Spain	Tarifa, Cadiz	1	Hpl E	KJ182953	Sánchez et al., 2014
*P. dioica*	Iceland	Krossavik	1	Hpl G	JN787102	Mols-Mortensen et al., 2012
	Britain	Aberystwyth, Sidmouth	2	Hpl J, G	AF081291, HQ687546	Klein et al., 2003; Sutherland et al., 2011
	Ireland	Spiddal	5	Hpl F	OL477663, OL477664, OL477665, OL477666, OL477667	This study (SPI)
		Salthill 2	4	Hpl F	OL477659, OL477660, OL477661, OL477662	This study (SAL2)
	France	Saint Malo	3	Hpl G	OL477668, OL477669, OL477670	This study (MAL)
	Spain	Herminia	3	Hpl G; I	OL477671, OL477672, OL477673	This study (HER)
		Esteiro	2	Hpl G; I	OL477674, OL477675	This study (EST)
	Portugal	Gelfa	4	Hpl H; G	OL477676, OL477677, OL477678, OL477679	This study (GEL)
		Buarcos	6	Hpl F; H	OL477680, OL477681, OL477682, OL477683, OL477684, OL477685	This study (BUA)
		Oeiras	2	Hpl F; K	OL477686, OL477687	This study (OEI)
W *P. dioica*	Portugal	Raso^ + +^	1	Hpl F	OL477695	This study (RAS)
		Amado	5	Hpl F	OL477688, OL477689, OL477690, OL47791, OL477692	This study (AMA)

*N: Number of individuals per sampling site, Hpl: Haplotype, Accession number in Genbank, Source (Reference used or individuals from a population from this study, for code in brackets refer to Table 1). ^++^Mixed individuals of P. linearis and Winter P. dioica in the same population.*

The cpDNA phylogroups observed for *P. linearis* (North Central Europe vs. South Europe) and the winter form of *P. dioica* matched remarkably well with the clusters observed for the microsatellites, as illustrated in the PCA plot (Figure 6B). In *P. linearis*, two main biogeographical areas were found: a larger area representing North-Central Europe in addition to one site from northern Spain, where only a single haplotype (A) was found, and a southern area with 6 haplotypes and evidence of introgression. For *P. dioica* (annual form) we also found six haplotypes, all very closely related (only one or two mutation steps difference among them), but there was no clear geographical structure in their distribution. However, also here, the South Europe cluster, with 5 haplotypes, showed more diversity than the North Central cluster, with only 3 haplotypes.

## Discussion

### Species Delimitations, Cryptic Diversity and the Winter Forms of *P. dioica*

The two species were well delimited by both the nuclear and chloroplast markers; in all analyses the observed groups/clusters were associated with the two species. Surprisingly, our analyses did reveal that one of the species, *P. dioica*, presented two morpho/ecotypes, an annual form and winter form indistinguishable from *P. linearis.* This winter form was found to co-exist with individuals of *P. linearis* but also to be the only form present at one site. No genetic differentiation was found between the winter form and the annual form of *P. dioica*, judging from the *F*_CT_ values between these two groups that were very close to zero. This suggests that the striking morphological differences between the forms could be the result of phenotypic plasticity, which is a genotype’s ability to produce variable phenotypes in response to environmental conditions (e.g., West-Eberhard, 2003). Phenotypic plasticity has previously been reported for other *Porphyra* species (e.g., Varela-Álvarez et al., 2007). Moreover, it is recognized, if not well known, that a single *Porphyra* species can present several morphological forms (e.g., linear, lanceolate, and/or rosette morphologies, see Reddy et al., 2018, for example). In addition, several species can share the same morphology; this is a well-known cryptic diversity issue in *Porphyra sensu lato* (e.g., Varela Álvarez, 2002). This “winter form” of *P. dioica* could be a plastic response of *P. dioica* to environmental conditions in the south. In contrast, the morphological differences between the two forms of *P. dioica* could be under genetic control, which would make these morphotypes differentiated ecotypes. How the species can adapt/modify its form and seasonality, is something that we cannot answer from the results of this study, but it is clear that two distinct forms are associated with the genome of *P. dioica*. In addition, admixed and hybrid individuals between the two species were found.

The phenomenon of algal species showing similar morphologies has previously been ascribed (e.g., Littler et al., 1983) to convergent adaptations to critical environmental factors. Littler and Littler (1980) postulate that the functional characteristics of seaweeds, such as photosynthesis, nutrient uptake, and grazer susceptibility, are related to form characteristics. For example, growth and morphogenesis in the green algae of the order Ulvales depends on the combination of regulative morphogenetic compounds released by specific associated bacteria (Weiss et al., 2017). All these factors can hypothetically influence morphogenesis of any blade of Bangiaceae and could be additional reasons why simple seaweed species have similar morphologies. Shifts in the environment drive both plastic and evolutionary responses in organisms (reviewed in Schaum and Collins, 2014). We have related in this study morphology and seasonality with specific genotypes, which can explain, at least partly, cryptic diversity in these species. Studies on the blade morphogenesis and environmental and genetic factors that influence the morphology of bladed Bangiaceae could also give important clues in resolving the conundrum of cryptic diversity.

### Biogeographical Patterns and Population Genetic Structure Detected

Though both clustering analyses (PCA and STRUCTURE) showed that the main divergence was at *K* = 2 for the distinction between the two species, there was also evidence of subclustering within each species (*K* = 3 and *K* = 4). For both *Porphyra* species, the microsatellite and chloroplast markers showed the presence of two main biogeographical areas within the Northeast Atlantic: North-Central Europe (including Iceland, Norway, Ireland, Britain, France, and Northeast Spain) and South Europe (Northwest Spain and Portugal). The degree of divergence between the northern and southern clusters, as measured using *F*_CT_, was stronger for *P. linearis* than for *P. dioica*, independent of the assumed ploidy level. For *P. linearis*, the southern regions hosted the maximum genetic variability and contained all cpDNA haplotypes including some unique to these areas and the Northern-central regions have fewer and non-unique haplotypes. For *P. dioica*, all the diversity seemed to be more mixed across its distribution with no clear pattern, though it was also lower in the north compared with the south in general. Additionally, at the more extreme south, introgressed *P. linearis* individuals and mixed populations with the winter form of *P. dioica* start to appear.

Combined, these results point to a postglacial recolonization from southern to northern Europe after the LGM (Last Glacial Maximum), where *P. linearis* probably suffered more from colonization bottlenecks or from extirpation events in the past followed by subsequent recolonization, which may have resulted in the high genetic differentiation between the two areas. In the case of *P. dioica*, the separation of the two biogeographical areas lies more to the north as the population from Brittany still clusters with the southern populations but the peripheral Irish populations form their own cluster. These populations in *P. dioica* seem to be genetically differentiated from the others suggesting reduced gene flow with other populations in Europe. The difference in patterns between the two species may reflect the differences in their life history; *P. dioica* is dioecious and is present all year round, *P. linearis* is protandrous hermaphroditic and only present in the winter and the spring, and therefore has higher demographic turnover rates. Our study lacks extensive sampling toward the North for both species, so maybe when more samples from this area would be available, the split between the northern and southern clusters may become more apparent. The southern area of Europe, including west Iberia, could have acted as a climatic refugium for both species, reflecting patterns of past glaciation in Iberia (Neiva et al., 2014; Bermejo et al., 2018) harboring most of the gene pool of each species. High genetic differentiation has been shown for populations of other red algal species in different geographical areas within Europe [e.g., *Mastocarpus stellatus* (Stackhouse) Guiry; Zuccarello et al., 2005] including non-interbreeding populations within the same putative species (Guiry and West, 1983). However, in the case of *P. dioica*, more recent historical events might hypothetically have led to admixture and connectivity among population, in addition to a possible role of different mating systems and all year presence on the shore (vs. only winter/spring presence in *P. linearis*).

### Evidence of Plastid Introgression and Admixed Populations at the South

The transfer of genetic material across species by hybridization and/or introgression has a considerable impact on the genetic make-up and evolution of species (e.g., Mallet, 2005; Arnold et al., 2008; Neiva et al., 2010). For other species of *Porphyra sensu lato*, the phenomena of hybridization and introgression have previously been observed (e.g., Niwa and Sakamoto, 2010; Niwa et al., 2010, 2018; Mols-Mortensen et al., 2012); however, these processes have not been put in a geographical context. Here, we showed evidence of plastid introgression and admixture within populations of *P. linearis*, with individuals having the genome of *P. linearis* but the chloroplast of *P. dioica* or other divergent chloroplast probably from another *Porphyra* species. Additionally, we found the winter form of *P. dioica*, which can appear between *P. linearis* patches or as independent patches/populations but in the same niche (high on the shore) as *P. linearis*. We observed such diversity only within the southern cluster of *P. linearis*, but never in the northern cluster nor in any population of *P. dioica*.

At the edge of their distributional ranges, some species may show distinct evolutionary patterns and processes (e.g., Budd and Pandolfi, 2010; Malay and Paulay, 2010), and our data indicate that this is a likely hypothesis for our study species. It would be interesting to know to what extent the introgression for *P. linearis* reported in the literature [*P. umbilicalis* Kützing and *P.* linearis Greville (Mols-Mortensen et al., 2012)] and cryptic diversity (Klein et al., 2003; Lindstrom and Fredericq, 2003; Kucera and Saunders, 2012; Mols-Mortensen et al., 2012) are associated with specific geographical areas (like in this study for southern Europe) or particular evolutionary processes.

### The Genetic Analyses of a Mixoploid Chimera

To devise a method to analyze genetic structure while incorporating alleles derived from two processes – blades with several genotypes in the same individual but also with different genome sizes and/or different cytotypes (ploidy levels) – has been challenging. Since these genotypes and ploidy levels can be either distributed across separate sections of the blade or distributed haphazardly (see Varela-Álvarez et al., 2021), it is not possible to know the exact ploidy level of a thallus portion used for genotyping *a priori* (or *a posteriori*). As the sample is used destructively for DNA extraction, the exact same slice cannot be used for measuring the ploidy level with flow cytometry. The most feasible way to analyze the data of a mixoploid plant was to resample the data sets several times. In that way we could look at the population differentiation simulating each ploidy level separately, and then compare their strength. Though the resulting values of the summary statistics will of necessity be imprecise, this method does allow us to judge whether the assumption of a specific ploidy level would have any impact on the results (Meirmans et al., 2018). Our analyses, considering the data as either diploid, tetraploid or octoploid derived from haploid, diploid or tetraploid chimeric thalli, revealed no large differences in either estimations of genetic diversity or clustering methods in any assumed ploidy level. This indicates that our main conclusions are not strongly influenced by the assumed ploidy level of the data.

In red algae, the number, size and position of nuclei in cells as well as their DNA content (ploidy) vary considerably across taxa. DNA measurements have revealed that nuclei within a single individual can become polygenomic (either polyploid or polytene) and can also represent different stages of the life cycle (e.g., arrest at the G2 phase of mitosis) (Goff and Coleman, 1990) or represent different genome types (Varela-Álvarez et al., 2021). Multiple copies of a locus (either from ploidy changes or endopolyploidy) can evolve by mutation and recombination and form new alleles even within the same cell. However, this is often overlooked in population genetic studies of red algal taxa. The impact that the presence of different ploidy levels within one individual (mixoploidy and/or endopolyploidy) has on the genetic diversity and evolutionary biology of these species is poorly understood. The genetic analyses of mixoploids (organisms with more than one ploidy level in the same individual) has barely been studied in the literature, making the present study rather novel. We hope the results of this study may provide a starting point for stimulating additional studies for other *Porphyra sensu lato* taxa or other intertidal red algae considering these facts.

## Conclusion

Two biogeographical areas in the Northeast Atlantic have been found for chimeric blades of the genus *Porphyra* with higher genetic diversity and evidence of introgression and cryptic plasticity in the southern populations. The southern cluster in *P. linearis* revealed introgressed individuals that had cpDNA-haplotypes of *P. dioica*, as well as a winter form of *P. dioica* that grows intermixed with and is morphologically indistinguishable from *P. linearis*. For the latter species, we hypothesized that a northward colonization from southern Europe after the LGM has led to more homogeneous and lower genetic diversity in populations toward the north. For *P. dioica*, the signature of such recolonization is less obvious, possibly due to a higher connectivity and gene flow among populations resulting from its reproductive mode and annual seasonality. We hope the results of this study may provide a starting point showing how to tackle methodological challenges for other Bangiaceae and other intertidal red algal species considering mixoploidy and chimerism.

## Data Availability Statement

The datasets presented in this study can be found in online repositories. The names of the repository/repositories and accession number(s) can be found in the article/Supplementary Material.

## Author Contributions

EV-Á conceived the initial idea following discussion with all authors. EV-Á, ES, and MG performed the field collections. EV-Á performed the lab work and wrote the first draft of the manuscript. EV-Á and PM analyzed the data. PM designed the customized R-Scripts used in this study. All authors assisted substantially with the manuscript development and also approved the submitted version.

## Conflict of Interest

The authors declare that the research was conducted in the absence of any commercial or financial relationships that could be construed as a potential conflict of interest.

## Publisher’s Note

All claims expressed in this article are solely those of the authors and do not necessarily represent those of their affiliated organizations, or those of the publisher, the editors and the reviewers. Any product that may be evaluated in this article, or claim that may be made by its manufacturer, is not guaranteed or endorsed by the publisher.

## Footnotes

1
http://popart.otago.ac.nz

## Supplementary Material

Supplementary Figure 1 Plot of ΔK statistic of Evanno et al. (2005) detecting the number of K groups that best fit the data (best *K* = 2), **(A)** with data as 4×; **(B)** with data as 8×.

Supplementary Figure 2 Genome constitution of the 442 genotypes inferred by STRUCTURE to 2, 3, and 4 inferred clusters based in 10 microsatellite loci, considering the data as 4×.

Supplementary Figure 3 Genome constitution of the 442 genotypes inferred by STRUCTURE to 2, 3, and 4 inferred clusters based in 10 microsatellite loci, considering the data as 8×.

Supplementary Figure 4 Principal component analyses (PCA) based on allelic variation at 10 loci, considering the data as **(A)** tetraploid 4× and **(B)** octoploid 8×. PCAs with *Porphyra linearis* genotypes in red (north Europe light red, south Europe dark red), *Porphyra dioica* genotypes in blue (central Europe dark blue, south Europe light blue), Winter form of *P. dioica* in blue surrounded by a black circle and hybrids or genotypes with combined genomes in gray.

Supplementary Appendix 1 Input file with the 442 genotypes used in this study.

Supplementary Appendix 2 R Script to run Structure with input file coded as 4×.

## Glossary

Allopolyploid: are polyploids with chromosomes derived from two or more diverged taxa.
Chimera: an individual blade with cells of different genetic constitution
Diploid: a genome containing two copies of each chromosome.
Haploid: a genome containing one copy of each chromosome.
Mixoploid blades: blades with different ploidy levels.
Polygenomy: Term used by Goff and Coleman (1990) to describe nuclei that could be polyploid or polytene (with polytene chromosomes).
Polytene chromosome: extra-large chromosomes consisting of multiple replicated DNA strands (chromatids).
Polyploid: a genome containing more than two homologous sets of chromosomes.
Tetrad: the four products of meiosis, these remain attached in the life history of some taxa, including *Porphyra*.
