# Supplementary figures and images for: Biogeographic Population Structure of Chimeric Blades of Porphyra in the Northeast Atlantic Reveals Southern Rich Gene Pools, Introgression and Cryptic Plasticity
Source: Front Plant Sci. 2022 Feb 24;13:818368. doi: 10.3389/fpls.2022.818368 (PMC8908385; doi:10.3389/fpls.2022.818368)

SUPPLEMENTARY FIGURE 1

A) As 4x

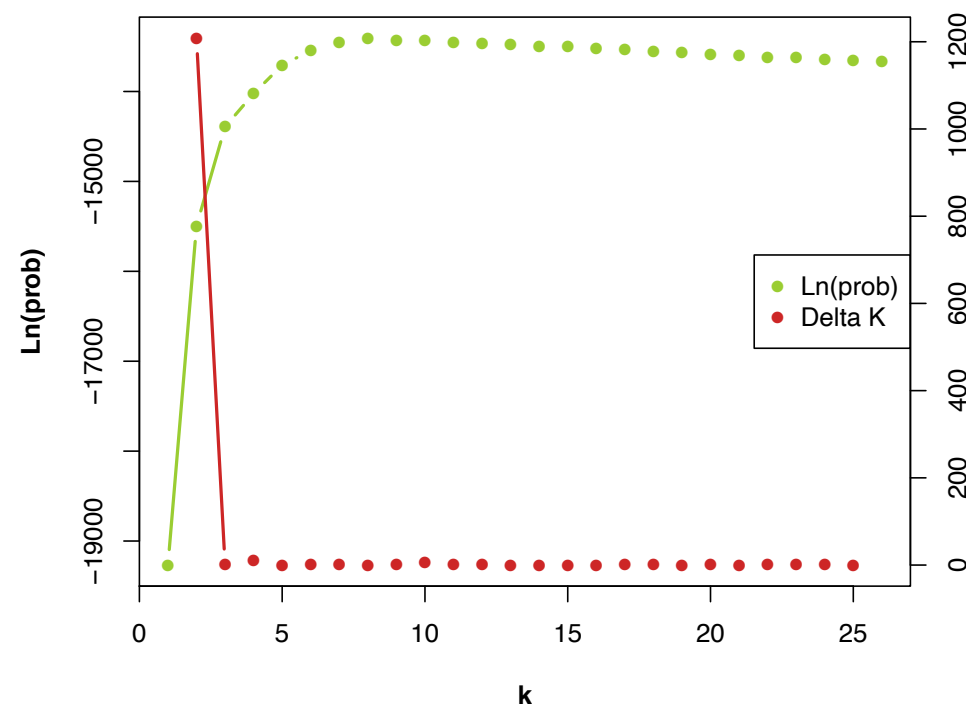

B) As 8x

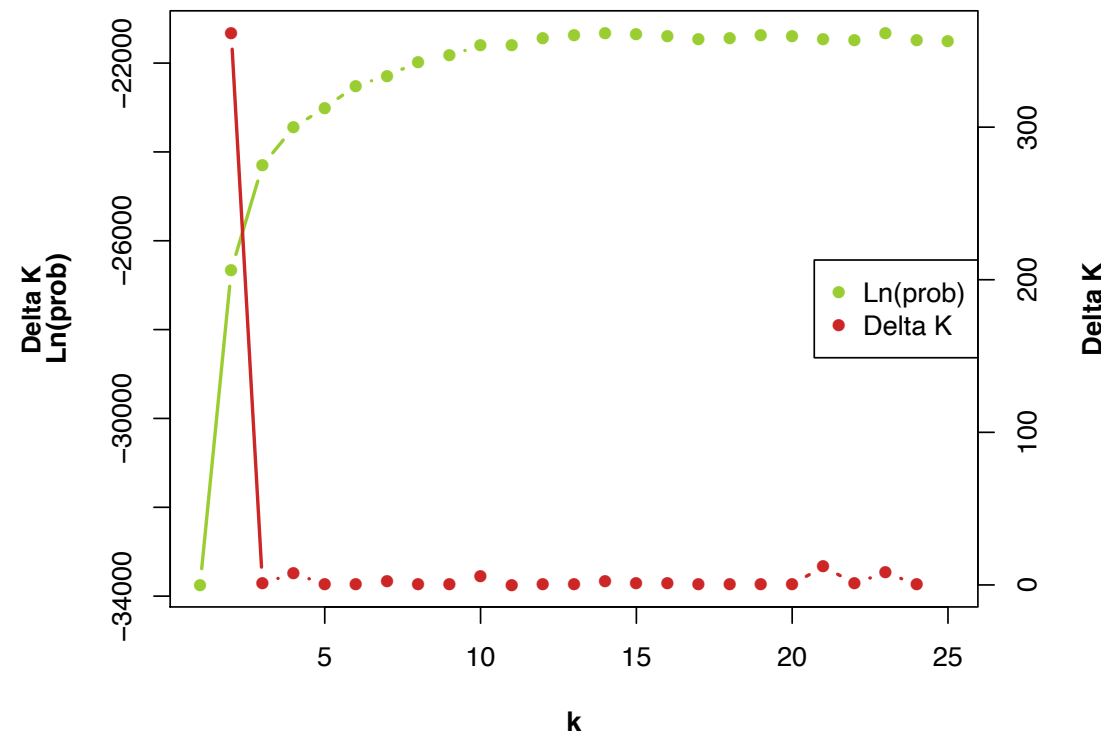

Supplement: Supplementary Figure 1 — Plot of ΔK statistic of Evanno et al. (2005) detecting the number of K groups that best fit the data (best K = 2), (A) with data as 4×; (B) with data as 8×. [file Image_1.pdf]

SUPPLEMENTARY FIGURE 2

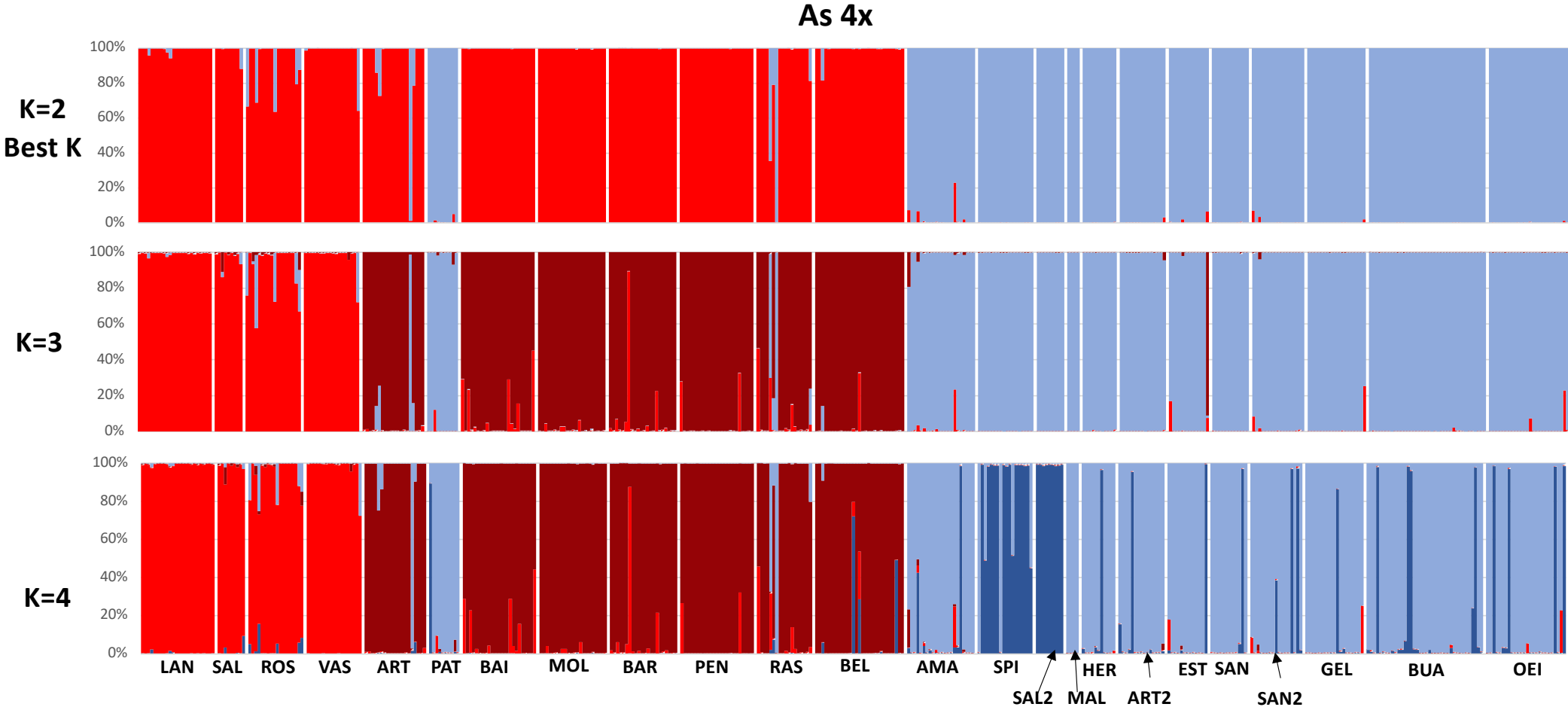

Supplement: Supplementary Figure 2 — Genome constitution of the 442 genotypes inferred by STRUCTURE to 2, 3, and 4 inferred clusters based in 10 microsatellite loci, considering the data as 4×. [file Image_2.pdf]

SUPPLEMENTARY FIGURE 3

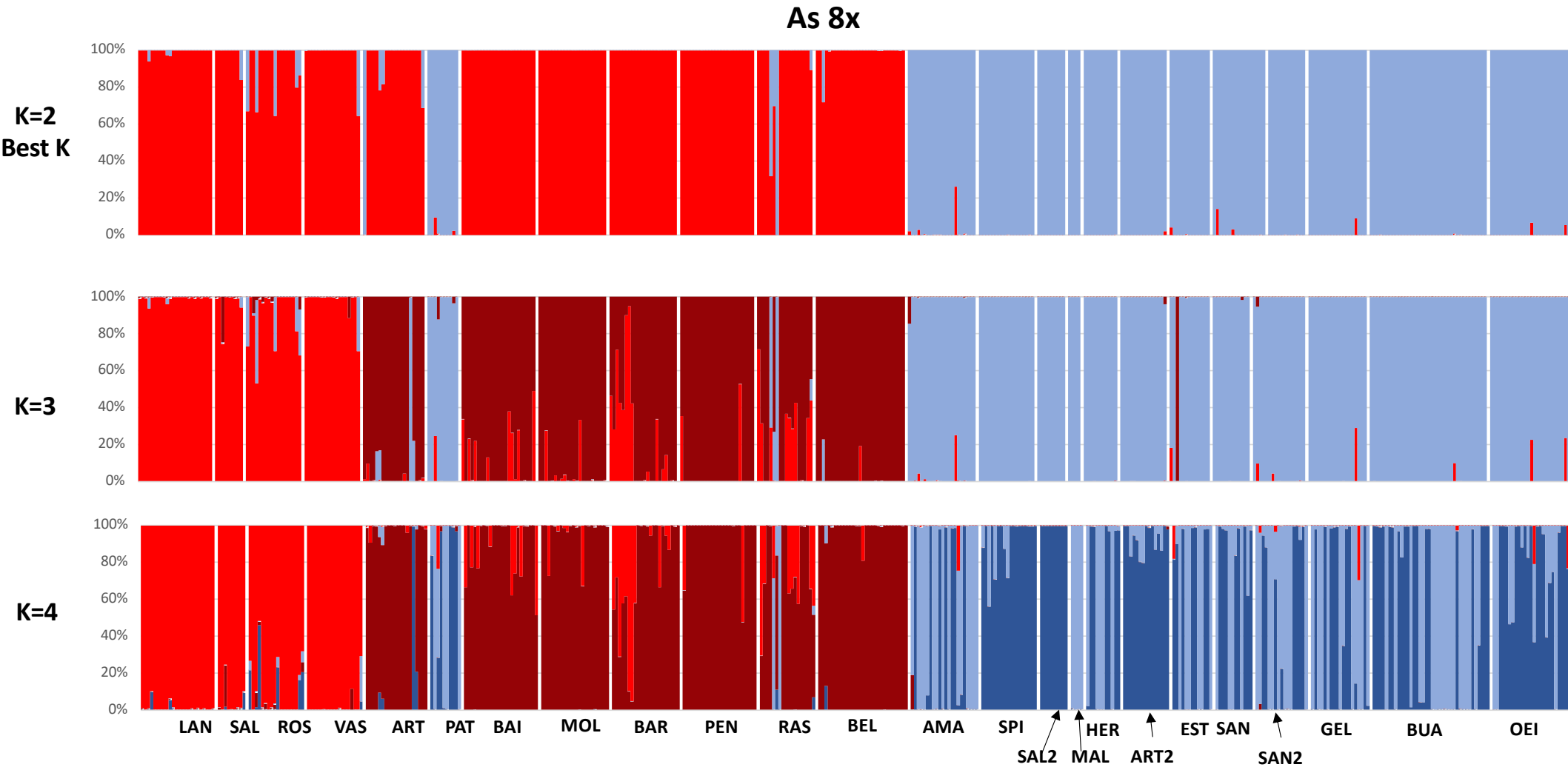

Supplement: Supplementary Figure 3 — Genome constitution of the 442 genotypes inferred by STRUCTURE to 2, 3, and 4 inferred clusters based in 10 microsatellite loci, considering the data as 8×. [file Image_3.pdf]

## SUPPLEMENTARY FIGURE 4

A) As 4x

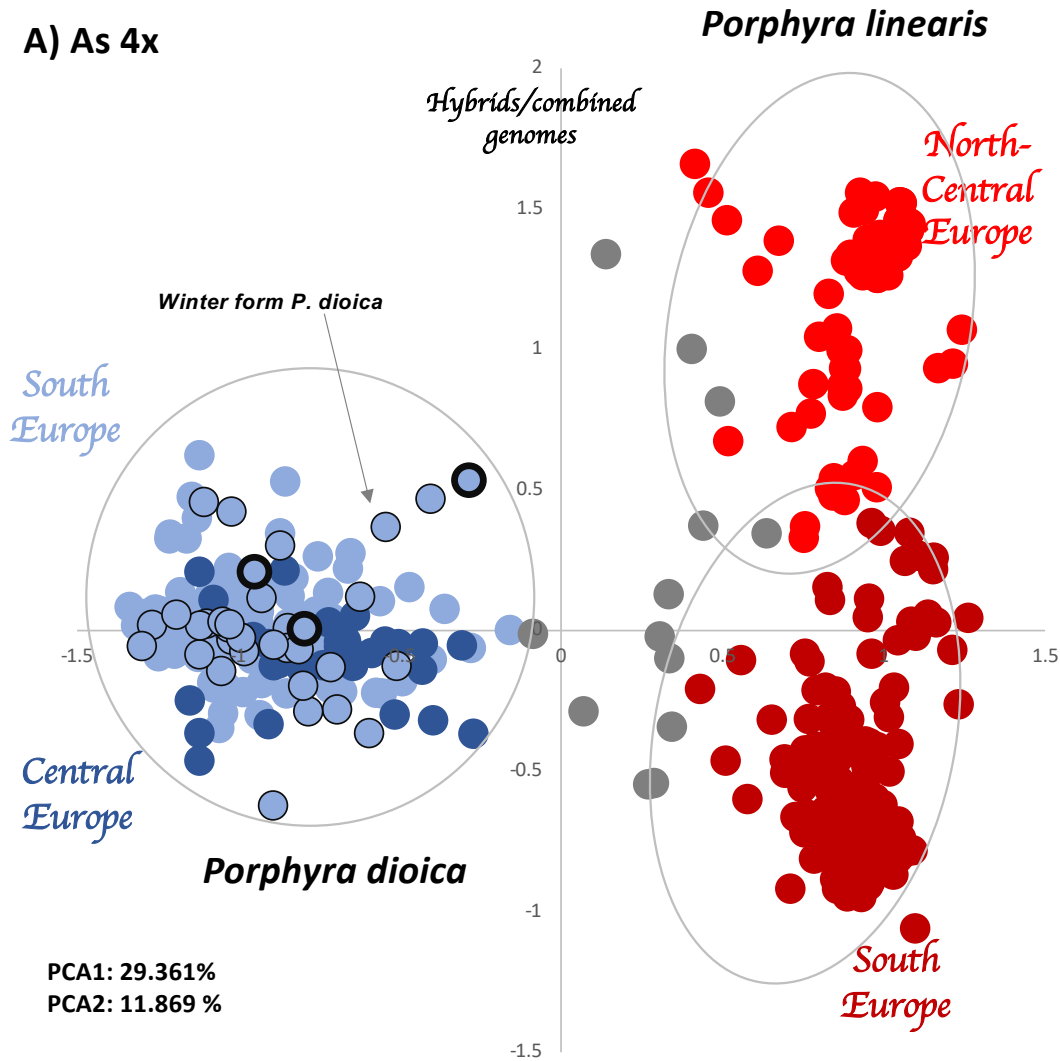

B) As 8x

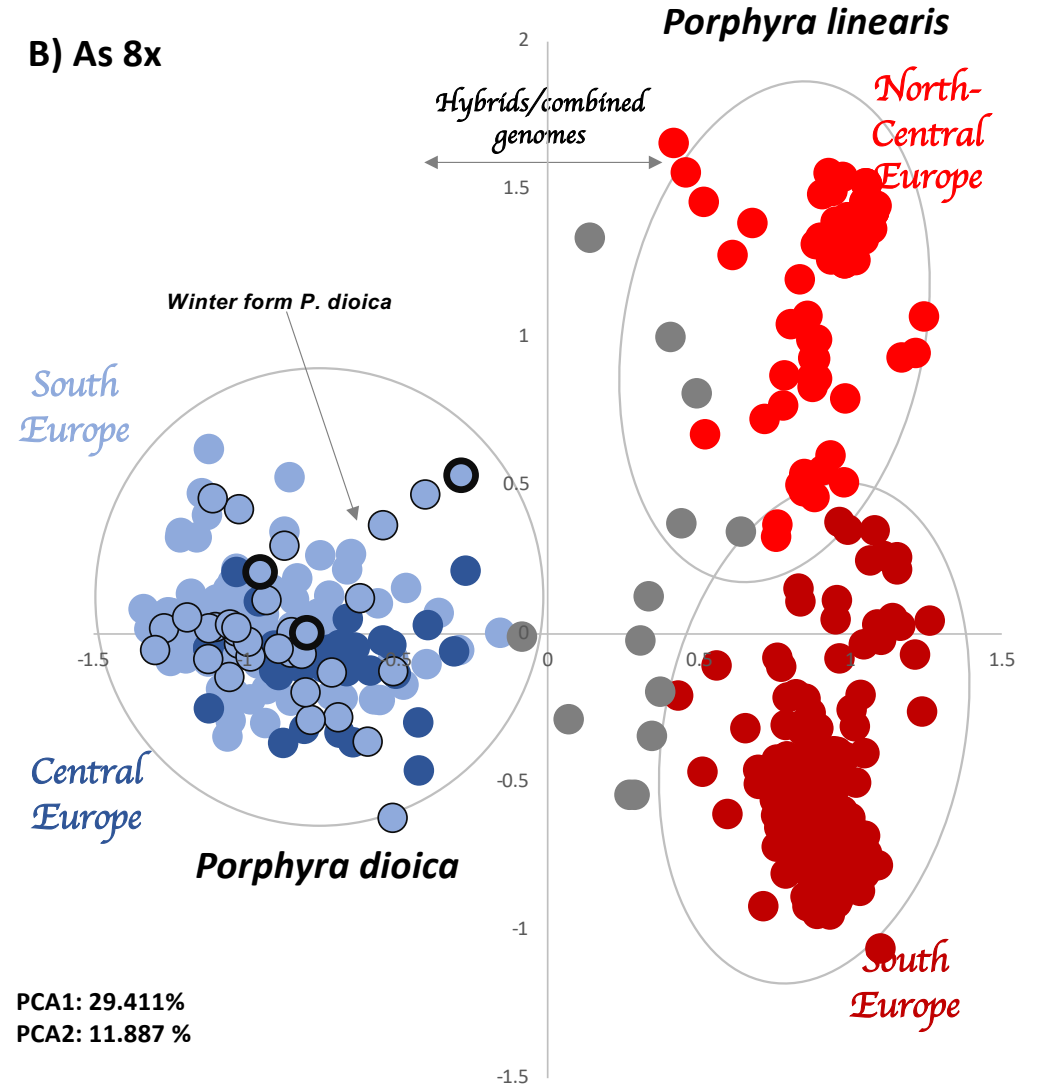

Supplement: Supplementary Figure 4 — Principal component analyses (PCA) based on allelic variation at 10 loci, considering the data as (A) tetraploid 4× and (B) octoploid 8×. PCAs with Porphyra linearis genotypes in red (north Europe light red, south Europe dark red), Porphyra dioica genotypes in blue (central Europe dark blue, south Europe light blue), Winter form of P. dioica in blue surrounded by a black circle and hybrids or genotypes with combined genomes in gray. [file Image_4.pdf]
