# Supplementary material for: Biogeographic Population Structure of Chimeric Blades of Porphyra in the Northeast Atlantic Reveals Southern Rich Gene Pools, Introgression and Cryptic Plasticity
Source: Front Plant Sci. 2022 Feb 24;13:818368. doi: 10.3389/fpls.2022.818368 (PMC8908385; doi:10.3389/fpls.2022.818368)
Supplement: Supplementary file 7 [file Table_1.docx]

**SUPPLEMENTARY TABLE 1:** PCR conditions of 10 microsatellite markers used in this study: Locus name, pcr conditions (Tª (C): annealing temperature/ PCR program: P1, P2, P3 / MgCl2 (mM): Magnesium concentration), and source (For details on the PCR program: P1, P2 and P3, see references).

| LOCUS NAME/ | Tª (C) /PCR/  MgCl_2_ (mM) | | Source |  |
| --- | --- | --- | --- | --- |
| PoLi_002 | 58 / P1 / 2 | Varela-Álvarez *et al*. 2017 | | |
| PoLi_004 | 56 / P3 / 1.8 | Varela-Álvarez *et al*. 2017 | | |
| PoLi_005 | 58 / P1 / 3 | Varela-Álvarez *et al*. 2017 | | |
| PoLi_006 | 58 / P1 / 2.5 | Varela-Álvarez *et al*. 2017 | | |
| PoLi_011 | 58 / P1 / 2.5 | Varela-Álvarez *et al*. 2017 | | |
| PoLi_012 | 58 / P1 / 2.5 | Varela-Álvarez *et al*. 2017 | | |
| PoLi_015 | 58 / P1 / 2 | Varela-Álvarez *et al*. 2017 | | |
| PoLi_017 | 58 / P1 / 2.5 | Varela-Álvarez *et al*. 2017 | | |
| PoLi_031 | 58 / P2/ 2.5 | Varela-Álvarez *et al*. 2017 | | |
| PoUm_012 | 56/P1/2 | Varela-Álvarez *et al*. 2018a | | |

Varela-Álvarez, E., Paulino, C., Serrão, E.A., 2017. Development and characterization of twelve microsatellite markers for *Porphyra linearis* Greville. *Genetica* 145: 127-130.

Varela-Álvarez, E., Balau, A., Paulino, C., Berecibar, E., Pearson, G., Serrão, E., 2018a. Isolation and characterization of microsatellite markers for the red alga *Porphyra umbilicalis*. *Plant Genetic Resources*, 1-4.
