# Supplementary material for: Biogeographic Population Structure of Chimeric Blades of Porphyra in the Northeast Atlantic Reveals Southern Rich Gene Pools, Introgression and Cryptic Plasticity
Source: Front Plant Sci. 2022 Feb 24;13:818368. doi: 10.3389/fpls.2022.818368 (PMC8908385; doi:10.3389/fpls.2022.818368)
Supplement: Supplementary file 8 [file Table_2.docx]

**SUPPLEMENTARY TABLE 2:** Pairwise genetic differentiation among all pairs of populations**.** Values of Nei´s Gst (above) vs. JostD (below) for all pair of populations (as 2x). All values P$\leq$0.001 except values in bold.

|  | **AMA** | **BEL** | **RAS** | **PEN** | **BAR** | **MOL** | **BAI** | **PAT** | **ART** | **VAS** | **ROS** | **SAL** | **LAN** | **OEI** | **BUA** | **GEL** | **SAN** | **SAN2** | **EST** | **ART2** | **HER** | **MAI** | **SAL2** | **SPID** |
| --- | --- | --- | --- | --- | --- | --- | --- | --- | --- | --- | --- | --- | --- | --- | --- | --- | --- | --- | --- | --- | --- | --- | --- | --- |
| **AMA** | 0 | 0.343 | 0.298 | 0.345 | 0.32 | 0.298 | 0.318 | 0.047 | 0.279 | 0.399 | 0.329 | 0.392 | 0.374 | 0.068 | 0.038 | 0.054 | 0.059 | 0.07 | 0.08 | 0.099 | 0.071 | 0.181 | 0.22 | 0.147 |
| **BEL** | 0.336 | 0 | 0.092 | 0.033 | 0.157 | 0.142 | 0.134 | 0.338 | 0.153 | 0.347 | 0.245 | 0.33 | 0.303 | 0.369 | 0.384 | 0.363 | 0.333 | 0.359 | 0.368 | 0.411 | 0.377 | 0.463 | 0.376 | 0.362 |
| **RAS** | 0.291 | 0.111 | 0 | 0.084 | 0.089 | 0.108 | 0.103 | 0.311 | 0.115 | 0.241 | 0.173 | 0.25 | 0.234 | 0.341 | 0.361 | 0.335 | 0.306 | 0.339 | 0.347 | 0.385 | 0.341 | 0.435 | 0.36 | 0.345 |
| **PEN** | 0.371 | 0.052 | 0.116 | 0 | 0.151 | 0.126 | 0.121 | 0.33 | 0.136 | 0.337 | 0.249 | 0.329 | 0.301 | 0.363 | 0.383 | 0.353 | 0.328 | 0.353 | 0.374 | 0.409 | 0.368 | 0.457 | 0.372 | 0.36 |
| **BAR** | 0.289 | 0.164 | 0.095 | 0.179 | 0 | 0.065 | 0.054 | 0.322 | 0.06 | 0.284 | 0.197 | 0.282 | 0.272 | 0.364 | 0.382 | 0.352 | 0.317 | 0.361 | 0.365 | 0.407 | 0.35 | 0.453 | 0.372 | 0.348 |
| **MOL** | 0.337 | 0.192 | 0.145 | 0.196 | 0.086 | 0 | **0.028** | 0.285 | **0.028** | 0.28 | 0.215 | 0.319 | 0.27 | 0.324 | 0.351 | 0.317 | 0.291 | 0.329 | 0.343 | 0.376 | 0.321 | 0.412 | 0.333 | 0.314 |
| **BAI** | 0.318 | 0.161 | 0.121 | 0.167 | 0.063 | 0.044 | 0 | 0.304 | 0.034 | 0.279 | 0.207 | 0.312 | 0.269 | 0.345 | 0.369 | 0.336 | 0.305 | 0.344 | 0.359 | 0.396 | 0.338 | 0.433 | 0.353 | 0.336 |
| **PAT** | **0.075** | 0.36 | 0.326 | 0.392 | 0.315 | 0.357 | 0.335 | 0 | 0.268 | 0.413 | 0.337 | 0.413 | 0.387 | 0.06 | 0.048 | **0.051** | **0.07** | 0.082 | 0.105 | 0.092 | **0.076** | 0.164 | 0.198 | 0.133 |
| **ART** | 0.308 | 0.193 | 0.144 | 0.198 | 0.075 | 0.048 | **0.05** | 0.327 | 0 | 0.298 | 0.223 | 0.326 | 0.286 | 0.309 | 0.333 | 0.299 | 0.274 | 0.312 | 0.323 | 0.359 | 0.302 | 0.397 | 0.329 | 0.304 |
| **VAS** | 0.472 | 0.43 | 0.33 | 0.465 | 0.346 | 0.42 | 0.378 | 0.519 | 0.417 | 0 | 0.075 | 0.118 | 0.106 | 0.427 | 0.459 | 0.433 | 0.393 | 0.429 | 0.455 | 0.47 | 0.449 | 0.509 | 0.438 | 0.445 |
| **ROS** | 0.363 | 0.294 | 0.215 | 0.33 | 0.224 | 0.298 | 0.259 | 0.401 | 0.292 | 0.166 | 0 | **0.094** | 0.065 | 0.349 | 0.371 | 0.353 | 0.318 | 0.35 | 0.37 | 0.4 | 0.383 | 0.442 | 0.361 | 0.355 |
| **SAL** | 0.384 | 0.34 | 0.267 | 0.375 | 0.275 | 0.367 | 0.326 | 0.43 | 0.356 | 0.222 | 0.149 | 0 | 0.073 | 0.422 | 0.438 | 0.433 | 0.398 | 0.431 | 0.451 | 0.454 | 0.455 | 0.528 | 0.432 | 0.419 |
| **LAN** | 0.415 | 0.36 | 0.289 | 0.395 | 0.303 | 0.369 | 0.332 | 0.458 | 0.367 | 0.236 | 0.126 | 0.13 | 0 | 0.391 | 0.416 | 0.404 | 0.369 | 0.396 | 0.424 | 0.435 | 0.426 | 0.498 | 0.406 | 0.4 |
| **OEI** | 0.107 | 0.388 | 0.355 | 0.421 | 0.351 | 0.394 | 0.372 | 0.108 | 0.367 | 0.533 | 0.417 | 0.443 | 0.467 | 0 | 0.04 | 0.042 | 0.088 | 0.088 | 0.12 | 0.044 | 0.093 | 0.157 | 0.156 | 0.09 |
| **BUA** | 0.051 | 0.348 | 0.32 | 0.381 | 0.316 | 0.361 | 0.338 | **0.071** | 0.334 | 0.489 | 0.378 | 0.397 | 0.426 | 0.063 | 0 | 0.042 | 0.068 | 0.073 | 0.082 | 0.074 | **0.075** | 0.15 | 0.193 | 0.115 |
| **GEF** | 0.08 | 0.363 | 0.33 | 0.392 | 0.323 | 0.367 | 0.345 | 0.085 | 0.338 | 0.511 | 0.396 | 0.425 | 0.452 | 0.074 | 0.06 | 0 | 0.045 | 0.052 | 0.07 | 0.08 | **0.051** | 0.143 | 0.209 | 0.133 |
| **SAN** | 0.073 | 0.304 | 0.272 | 0.331 | 0.264 | 0.305 | 0.284 | 0.095 | 0.279 | 0.435 | 0.328 | 0.359 | 0.382 | 0.12 | 0.079 | 0.06 | 0 | 0.033 | **0.05** | 0.13 | **0.04** | 0.194 | 0.22 | 0.166 |
| **SAN2** | 0.102 | 0.363 | 0.335 | 0.395 | 0.33 | 0.378 | 0.352 | 0.132 | 0.35 | 0.514 | 0.397 | 0.427 | 0.45 | 0.144 | 0.1 | 0.083 | **0.045** | 0 | **0.057** | **0.122** | **0.049** | 0.223 | 0.239 | 0.182 |
| **EST** | 0.094 | 0.317 | 0.291 | 0.353 | 0.285 | 0.333 | 0.31 | 0.132 | 0.305 | 0.463 | 0.354 | 0.38 | 0.406 | 0.153 | 0.091 | 0.088 | **0.054** | **0.073** | 0 | **0.146** | **0.053** | 0.241 | 0.281 | 0.223 |
| **ART2** | 0.138 | 0.395 | 0.364 | 0.431 | 0.358 | 0.41 | 0.385 | 0.145 | 0.383 | 0.535 | 0.43 | 0.44 | 0.473 | 0.077 | 0.101 | 0.122 | 0.157 | 0.177 | 0.168 | 0 | 0.111 | 0.221 | 0.216 | 0.147 |
| **HER** | 0.085 | 0.323 | 0.288 | 0.351 | 0.277 | 0.32 | 0.299 | 0.1 | 0.293 | 0.462 | 0.363 | 0.383 | 0.409 | 0.124 | 0.084 | **0.066** | **0.044** | **0.064** | **0.055** | 0.134 | 0 | 0.216 | 0.26 | 0.195 |
| **MAI** | 0.353 | 0.573 | 0.539 | 0.623 | 0.52 | 0.598 | 0.556 | 0.381 | 0.566 | 0.747 | 0.624 | 0.642 | 0.681 | 0.373 | 0.292 | 0.322 | 0.332 | 0.433 | 0.371 | 0.431 | 0.349 | 0 | 0.321 | 0.239 |
| **SAL2** | 0.319 | 0.438 | 0.411 | 0.478 | 0.397 | 0.451 | 0.422 | 0.331 | 0.429 | 0.601 | 0.477 | 0.499 | 0.531 | 0.285 | 0.277 | 0.325 | 0.291 | 0.359 | 0.336 | 0.336 | 0.32 | 0.639 | 0 | **0.054** |
| **SPID** | 0.208 | 0.387 | 0.361 | 0.424 | 0.344 | 0.391 | 0.369 | 0.214 | 0.367 | 0.549 | 0.425 | 0.445 | 0.478 | 0.159 | 0.162 | 0.203 | 0.208 | 0.262 | 0.254 | 0.222 | 0.231 | 0.484 | **0.123** | 0 |
